# The Story of the Insane from Year to Year

**Published:** 1901-05-04

**Authors:** 


### THE STORY OF THE INSANE FROM YEAR TO YEAR.

(Thirteenth Series.)

NORTHAMPTON COUNTY ASYLUM.

On January 1st, 1899, there were 918 patients in residence, and at the end of the year the number was reduced to 890; but this reduction was caused by the transfer of chronic patients chargeable to other counties, and not to any decrease in the admissions from Northamptonshire. During the year there were 222 first admissions: 25 readmissions, 59 recoveries, 25 discharged relieved, and 93 deaths. The recoveries stand at 31·4 per cent. of the admissions, and the deaths at 10·2 per cent. on the average number resident. Of the deaths 37 were caused by cerebral and spinal diseases, 15 by phthisis, 5 by pneumonia, and 1 by enteritis. Of the year's admissions 10 owed their insanity to various moral causes, 58 to hereditary tendency, 23 to intemperance in drink, and 58 were of unknown origin. The committee of visitors prepared a scheme whereby out-patients who could not afford to consult a private physician in mental diseases might attend at Berry Wood and obtain Dr. Harding's advice. This sensible innovation has been taken advantage of by several patients; but Dr. Harding thinks it will be "some time before they can be brought to recognise fully that an asylum is something more than a House of Detention," and he adds that "it is unfortunate that the name of asylum is applied to institutions whose primary object is the cure of disease." Well, there is something in a name, and why not follow the example of Dorset and call Berry Wood an "hospital"? We learn that the instruction classes for attendants and nurses are being continued by the recently appointed assistant medical officers (Messrs. Jones and Stuart), and that eight men and eight women have obtained the famous three-year Berry Wood certificate. The visitors say they feel "that there is a not unreasonable desire on the part of the friends of private patients that they should have accommodation apart from the pauper patients," and we trust we may interpret this to mean that the day is not far off when such accommodation will be provided. Such a step would relieve the anxiety felt by many residents in Northamptonshire who are quite unable to pay the charges made at private institutions, and it would fill the coffers of the asylum. The weekly charge to the unions is 7s. 6d. per head.

THE LAWN HOSPITAL FOR THE INSANE, LINCOLN.

On January 1st, 1899, the Lawn contained 81 patients, and at the end of December 80 were in residence. There were 28 admissions: 14 recoveries, 3 discharged relieved, 7 not improved, and 5 deaths. The recoveries, calculated on the admissions, stand at the very high percentage of 56 and the deaths calculated on the average number resident stand at 6 per cent.; but, as we have formerly remarked in these notices the statistics of a single year are not of much value, though in this hospital the average percentage of recoveries during the last decade has been high and the percentage of deaths low. The deaths were all among the men, and were all caused by general paralysis and other brain diseases. Nothing seems to have occurred during the year to disturb the harmony of the institution, so far at least as the patients were directly concerned. There was no epidemic disease, and no patient had to be restrained or secluded. Upwards of half of the patients attend the associated entertainments, and a large party were taken to the seaside under care of the assistant medical officer. It is much to be regretted that the lady assistant medical officer resigned during the year, and the committee have very wisely decided to increase the salary attached to the office, and have also raised the wages of the attendants and nurses. The committee say they have admitted several cases at reduced rates, and that the rates of some of the other inmates have been reduced. We do not see any table showing the sums at which patients are maintained. The outstanding debt has been considerably reduced, and it is much to be desired that a few rich and charitably disposed people would put their spare cash together and wipe it off entirely. We have not much hope that this will be done, for even the most charitable people in the kingdom will give to anything rather than to an institution for lunatics. Last year the Lawn received £41 16s. in subscriptions, but not a single legacy or donation was forthcoming; and we look upon this absence as a very discreditable fact in connection with a hospital that is trying to relieve those stricken with the saddest of all sad afflictions.

THE PORTSMOUTH BOROUGH ASYLUM.

There were in residence on the first day of 1899 748 patients, and on December 31st this number had been reduced to 689. There were 168 first admissions, 38 re-admissions, 112 recoveries, 51 discharged relieved, and 99 deaths. The recoveries were 56·56 per cent. of the admissions, and the deaths were 13·6 per cent. of the average number resident, both of these being high, the recoveries particularly so. Of the deaths 26 were caused by cerebral and spinal diseases, 17 by consumption, 11 by pneumonia, 3 by bronchitis, and 7 by colitis and dysentery. In addition to the 17 deaths from pulmonary consumption there were 7 from other forms of tubercular disease. The usual Table X. showing the causes of insanity in the year's admissions is not given. Dr. Mumby does not attempt any explanation of the high mortality from tubercular diseases, nor whether any steps are being taken to reduce it. The senior assistant medical officer resigned, and the post is now filled by Dr. Elliott, Dr. Barnes being the junior assistant. The clerk of the asylum (Mr. Woodeson) retired after 18 years' service and received, apparently, the highest pension allowed by the Act. The visitors say "that the officials, from the medical officer and his staff through all ranks, deserve their thanks for the work so satisfactorily carried out." The weekly charge for paupers is 10s. 6d. a head for those belonging to the borough, 14s. to 18s. for those chargeable to other counties or boroughs. Private patients pay from 14s. to 42s.

### THE IDIOT ASYLUM AT STARCROSS, EXETER.

THE number of patients in this asylum on January 1st, 1899, was 250, and the number on December 31 was also 250. There were 31 admissions, 2 recoveries, 15 much improved, 9 moderately improved, 2 not improved, and 3 died. In county lunatic asylums the male patients are generally fewer than the women; but in idiot asylums the reverse is the case; and Mr. Locke accounts for the fact by surmising "that girls, if not very deficient in intelligence, are more amenable to treatment at home, and that a certain amount of training may be practicable under a judicious mother." No doubt this is so, and it should also be remembered that the idiotic daughter may be useful in the house, and that the idiotic son is not only useless, but worse than useless. It is wonderful what can be done in teaching these idiot children if the deficiency of brain power be not too marked. At Starcross 32 of the boys are engaged in shoemaking, 40 in tailoring, 38 in basket-making, 21 in mat-making, and many others in carpentering, fretwork, gardening, &c. The girls find occupation in the laundry, sewing-room, kitchen, &c. Reading, writing, and arithmetic are also taught to those capable of learning, and we notice that 90 can read well, and 92 can write in copybooks. Some of the patients are admitted free or at reduced charges, but a table giving the various sums paid would be a desirable addition to the next report. The asylum is managed by a committee, of which the Earl of Devon is chairman. Mr. Locke is superintendent and secretary, and Mr. Lipscombe is medical officer.

### THE EAST SUSSEX ASYLUM AT HAYWARD'S HEATH.

THIS asylum contained 907 patients at the beginning of the year and 955 at the end of it. There were 227 first admissions, 44 re-admissions, 85 recoveries, 27 discharged relieved, and 90 deaths. The recoveries were 33·7 per cent. of the admissions, and the deaths were 9·6 per cent. of the average number resident. Of the deaths 51 were caused by cerebral and spinal diseases, 5 by consumption, 8 by pneumonia, and 3 by bronchitis. Moral causes account for the insanity in 22 of the admissions, intemperance in drink for 28, and hereditary influence for 68. Lectures to the staff were given by Dr. Walker to prepare the members for the examination of the Medico-Psychological Association; and 8 nurses and 13 attendants obtained the nursing certificate. With such a good beginning we trust the medical officers will see their way to institute a three years' course of instruction and a Hayward's Heath certificate. Dr. Saunders has, owing to ill health, had extended leave of absence, and has, we are pleased to learn, recovered his health and returned to duty. During his absence Dr. Walker acted as deputy; and the committee say they "have every reason to be satisfied with the general work of Dr. Saunders, his medical colleagues, and the officers generally." The weekly cost of maintenance was 9s. 4d. per head.

